# Loss of Cannabinoid Receptor CB1 Induces Preterm Birth

**DOI:** 10.1371/journal.pone.0003320

**Published:** 2008-10-03

**Authors:** Haibin Wang, Huirong Xie, Sudhansu K. Dey

**Affiliations:** Departments of Pediatrics, Cell & Developmental Biology, and Pharmacology, Vanderbilt University Medical Center, Nashville, Tennessee, United States of America; University of California San Francisco, United States of America

## Abstract

**Background:**

Preterm birth accounting approximate 10% of pregnancies in women is a tremendous social, clinical and economic burden. However, its underlying causes remain largely unknown. Emerging evidence suggests that endocannabinoid signaling via cannabinoid receptor CB1 play critical roles in multiple early pregnancy events in both animals and humans. Since our previous studies demonstrated that loss of CB1 defers the normal implantation window in mice, we surmised that CB1 deficiency would influence parturition events.

**Methods and Findings:**

Exploiting mouse models with targeted deletion of *Cnr1*, *Cnr2* and *Ptgs1* encoding CB1, CB2 and cyclooxygenase-1, respectively, we examined consequences of CB1 or CB2 silencing on the onset of parturition. We observed that genetic or pharmacological inactivation of CB1, but not CB2, induced preterm labor in mice. Radioimmunoassay analysis of circulating levels of ovarian steroid hormones revealed that premature birth resulting from CB1 inactivation is correlated with altered progesterone/estrogen ratios prior to parturition. More strikingly, the phenotypic defects of prolonged pregnancy length and parturition failure in mice missing *Ptgs1* were corrected by introducing CB1 deficiency into *Ptgs1* null mice. In addition, loss of CB1 resulted in aberrant secretions of corticotrophin-releasing hormone and corticosterone during late gestation. The pathophysiological significance of this altered corticotrophin-releasing hormone-driven endocrine activity in the absence of CB1 was evident from our subsequent findings that a selective corticotrophin-releasing hormone antagonist was able to restore the normal parturition timing in *Cnr1* deficient mice. In contrast, wild-type females receiving excessive levels of corticosterone induced preterm birth.

**Conclusions:**

CB1 deficiency altering normal progesterone and estrogen levels induces preterm birth in mice. This defect is independent of prostaglandins produced by cyclooxygenase-1. Moreover, CB1 inactivation resulted in aberrant corticotrophin-releasing hormone and corticosterone activities prior to parturition, suggesting that CB1 regulates labor by interacting with the corticotrophin-releasing hormone-driven endocrine axis.

## Introduction

Anandamide and 2-arachidonoylglycerol are two major endocannabinoids that activate two G protein-coupled cannabinoid receptors CB1 and CB2, encoded by *Cnr1* and *Cnr2*, respectively [1]–[4]. These endocannabinoids mimic many effects of Δ^9^-tetrahydrocannabinol (THC), a major psychoactive component of marijuana, on central and peripheral systems [5]–[10]. With respect to female reproduction, we first provided evidence in mice for the presence of CB1 in preimplantation embryos [11], [12] and of anandamide in the oviduct and uterus [12]–[15], suggesting that endocannabinoid signaling is operative during early pregnancy. This was further supported by our findings of biphasic effects of anandamide on embryo development and implantation [16], [17]. For example, anandamide at a low concentration makes the blastocyst competent for implantation, while at a higher concentration it attenuates this response [16]. These observations later led to studies in humans showing an association of higher anandamide levels with spontaneous pregnancy loss [18]. Moreover, we recently found that loss of CB1 often derails oviductal embryo transport, preventing on-time implantation [19]. Since a short delay in on-time implantation adversely affects later developmental processes [20]–[22], we explored the consequences of silencing of CB1 on the onset of labor.

Here we demonstrate that CB1 inactivation in mice induces preterm labor by altering normal progesterone/estrogen ratios prior to parturition. This defect is independent of cyclooxygenase-1 (COX-1)-produced prostaglandins, since loss of CB1 overrides delayed parturition that occurs in *Ptgs-1* deficient mice. More strikingly, loss of CB1 resulted in altered corticotrophin-releasing hormone (CRH) and corticosterone (CTS) levels during late gestation, suggesting that CB1 regulates labor by interacting with CRH-CTS endocrine axis. This study has high clinical relevance, since there is evidence that normal onset and duration of labor involve anandamide signaling in women [23], [24] and that CRH is considered as a pregnancy clock controlling pregnancy length in women [25], [26]. This is an issue of concern, since preterm birth accounting approximate 10% of pregnancies in women is a major social, clinical and economic burden [27], [28].

## Results and Discussion

### CB1 deficiency induces preterm labor in mice

**Figure 1:**
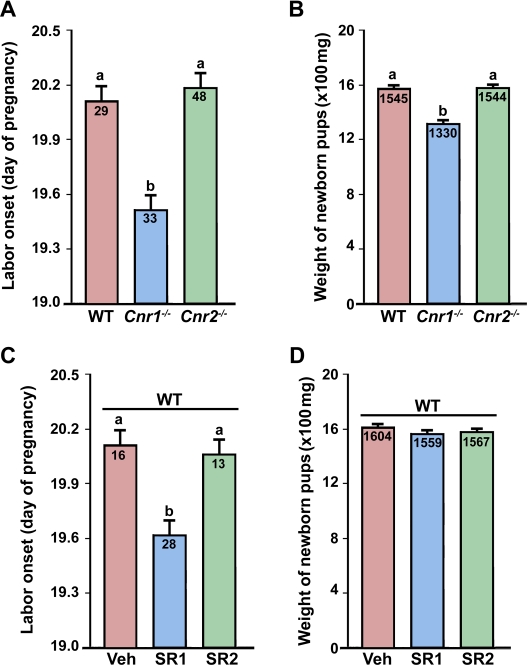
Genetic or pharmacological silencing of CB1 induces preterm labor. (A & B) Mice missing *Cnr1*, but not *Cnr2*, show early onset of labor with reduced fetal weights at birth. (C & D) Preterm labor occurs in wild-type (WT) pregnant mice receiving a CB1-selective antagonist SR141716 (SR1), but not a CB2-selective antagonist SR144528 (SR2), on days 14–18 with little effects on fetal weights. Numbers within bars indicate the number of mice examined in panels A and C. The average fetal weights (mg) at birth are shown in panels B and D. The bars with different letters are significantly different (P<0.01).

Increasing evidence points toward critical roles of endocannabinoid signaling during early pregnancy [8]–[10]. We recently demonstrated that loss of CB1 derails oviductal embryo transport, leading to deferral of on-time embryo implantation [19]. Since an initial deferral of implantation is often associated with delayed parturition [22], we speculated that CB1 deficiency would result in delayed parturition in mice. However, we observed that genetic loss of *Cnr1*, but not *Cnr2*, leads to preterm labor (**Figure 1A**). Consequently, fetal weight at birth in *Cnr1* ^−/−^ mice is significantly reduced compared with wild-type (WT) mice (**Figure 1B**). In contrast, the parturition events in mice missing *Cnr2* are apparently normal (**Figure 1A & B**).These results may explain why *Cnr1* ^−/−^ pups display poor suckling activities during early days after birth and why their weight gain during postnatal development remains significantly lower than WT control pups [29].

Since *Cnr1* ^−/−^ mice display multiples defects during early pregnancy, including asynchronous preimplantation embryo development [12], impaired oviductal embryo transport and deferred implantation [19], it was not clear whether preterm birth in *Cnr1* ^−/−^ mice was seeded during early pregnancy or resulted from CB1 deficiency during the late gestational period. Therefore, we next examined the consequences of silencing cannabinoid signaling in WT mice by subcutaneous administration of selective CB antagonists during late gestation. As illustrated in **Figure 1C & D**, similar preterm parturition occurred in WT mice receiving a CB1-selective antagonist SR141716 (SR1) [30], but not a CB2-selective antagonist SR144528 (SR2) [31], on days 14–18 of pregnancy. These observations suggest that endocannabinoid signaling via CB1 is critical to normal gestational length.

The initiation of parturition results from a synchronous interplay of both maternal and fetal factors [28]. Thus, we assessed the contribution of maternal versus embryonic CB1 to parturition defects in *Cnr1* ^−/−^ mice by mating null females with wild-type males to generate all heterozygous embryos. We observed that preterm labor phenotype was sustained in pregnant *Cnr1* ^−/−^ females bearing heterozygous embryos, although birth weights of heterozygous pups were comparable to those of wild-type pups at birth (**Figure S1A & B**). The results indicate that maternal CB1 signaling is critical for timely onset of labor.

### CB1 deficiency alters normal progesterone and estrogen secretions prior to parturition in mice

**Figure 2:**
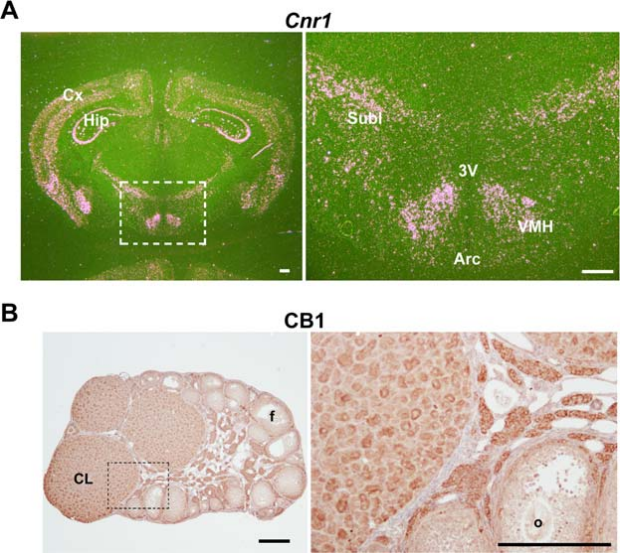
Expression of CB1 transcript and protein in the wild-type (WT) brain (A) and ovary (B) on day 18 of pregnancy. Bar, 100 µm. 3V, third ventricle; Arc, arcuate nucleus of the hypothalamus; Cx, cortex; Hip, hippocampus; Subl, subincertal nucleus; VMH, ventromedial hypothalamic nucleus; CL, corpus luteum; f, follicle; o, oocyte.

**Figure 3:**
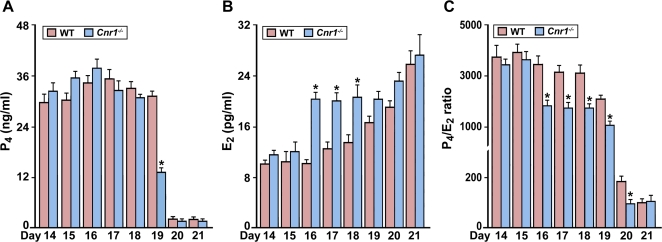
CB1 deficiency alters normal progesterone (P_4_) and estradiol-17β (E_2_) levels prior to parturition in mice. Serum P_4_ and E_2_ levels were analyzed by radioimmunoassay. While CB1 deficiency induced an early drop in serum P_4_ levels on day 19 (A), circulating E_2_ levels were elevated on days 16–18 (B), resulting in a remarkable decrease in P_4_/E_2_ ratio prior to labor in *Cnr1* ^−/−^ females (C) (n = 6–10, *P<0.05).

Recent evidence suggests the involvement of endocannabinoid signaling via central CB1 in neuroendocrine regulation of reproduction. For example, both exogenous cannabinoids and endocannabinoids have been shown to modulate the secretion of hypothalamic and pituitary hormones including luteinizing hormone and prolactin in rodents [32]–[36]. To reveal potential causes of preterm birth in the absence of CB1 in mice, we first examined the expression of CB1 in the hypothalamus and ovary. As illustrated in **Figure 2A & B**, we observed a wide distribution of CB1 in these tissues on day 18 of pregnancy, suggesting the contention that endocannabinoids would impact the hypothalamic-ovarian axis at multiple levels during late gestation. Since functional progesterone (P_4_) withdrawal either due to fall in circulating P_4_ levels or attenuation of P_4_ action together with heightened estrogen action determines the parturition timing in most viviparous species including humans [37], we measured serum levels of P_4_ and 17β-estradiol (E_2_) in *Cnr1* ^−/−^ females during late gestation to assess potential causes of preterm labor in the absence of CB1. As shown in **Figure 3A**, CB1 deficiency induced an early drop in serum P_4_ levels on day 19 of pregnancy. In contrast, circulating E_2_ levels substantially increased on days 16–18 with the loss of CB1 (**Figure 3B**). This inverse relationship between P_4_ and E_2_ levels in the absence of CB1 creates a significant decrease in P_4_/E_2_ ratio (**Figure 3C**), leading to preterm birth in *Cnr1* ^−/−^ females.

**Figure 4:**
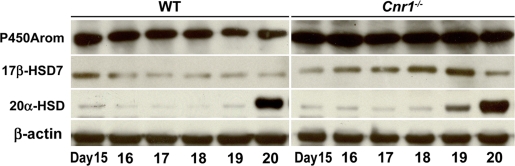
Western blot analysis of cytochrome P450 aromatase (P450Arom), 17β-hydroxysteroid dehydrogenase 7 (17β-HSD7) and 20α-HSD in wild-type (WT) and *Cnr1^−/−^* ovaries during late gestation.

Western blotting analysis of key steroid biosynthetic and metabolic enzymes demonstrated that while levels of cytochrome P450 cholesterol side-chain cleavage enzyme and 3β-hydroxysteroid dehydrogenase (3β-HSD) were comparable in wild-type and *Cnr1* null ovaries (data not shown), levels of cytochrome P450 aromatase (P450Arom) and 17β-HSD7, which primarily contribute to ovarian E_2_ biosynthesis during gestation in mice [38], were upregulated in *Cnr1^−/−^* ovaries (**Figure 4**). Moreover, levels of 20α-HSD, which metabolizes P_4_ into biologically inactive 20α-dihydroprogesterone, were substantially increased in *Cnr1^−/−^* ovaries on day 19 of pregnancy as opposed to that occurs in WT ovaries on day 20 (**Figure 4**). These temporal changes in P_4_ metabolic and estrogen biosynthetic enzymes in null ovaries correlate well with our finding of early fall in P_4_ with rising E_2_ levels preceding early onset of parturition. As shown in **Figure S2A & B**, our observation of restoration of normal parturition in *Cnr1^−/−^* mice by a subcutaneous injection of P_4_ (1 mg/mouse) on day 18 further supports that a decreased P_4_/E_2_ ratio is a cause of preterm labor in null females. With respect to contribution of gonadotropins and prolactin on this altered ovarian P_4_ and E_2_ secretion pattern in the absence of CB1, similar circulating levels of luteinizing hormone were observed in WT and *Cnr1^−/−^* mice on days 14–18 of pregnancy. However, basal levels of follicle stimulating hormone substantially increased in *Cnr1^−/−^* mice (data not shown). Placenta-derived prolactin-like hormones, but not pituitary prolactin, primarily act to maintain luteal P_4_ secretion during late gestation in mice [38], [39]. Nonetheless, it would be interesting to see in future studies whether CB1 deficiency alters prolactin secretion at various stages of pregnancy.

### Loss of CB1 overrides delayed parturition that occurs in *cyclooxygenase-1* deficient mice

**Figure 5:**
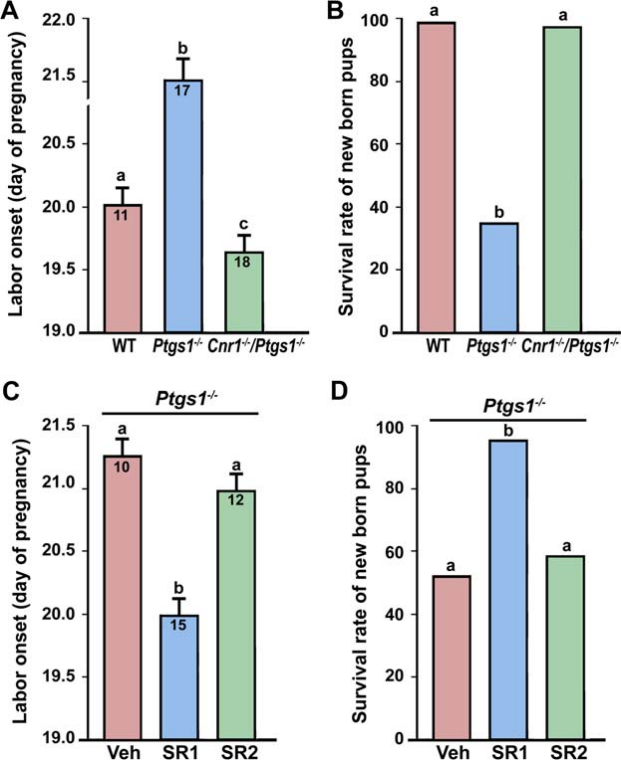
Loss of CB1 overrides delayed parturition that occurs in *Ptgs1^−/−^* mice. (A & B) The onset of parturition and survival rate of newborn pups in mice missing both *Cnr1* and *Ptgs1* are substantially improved in contrast to delayed parturition in *Ptgs1^−/−^* mice. (C & D) Parturition defects were largely restored in pregnant *Ptgs1^−/−^* mice receiving a CB1-selective antagonist SR141716 (SR1), but not a CB2-selective antagonist SR144528 (SR2), on days 14–18. Numbers within the bars indicate number of mice examined in panels A and C. The bars with different letters are significantly different (P<0.01).

In mice, genetic ablation of *Ptgs1*, encoding cyclooxygenase (COX)-1, results in delayed or failure of parturition because of impaired luteolysis with sustained P_4_ production [40], [41]. To confirm our finding that early decline in serum P_4_ levels is a trigger for preterm birth in *Cnr1^−/−^* mice, we introduced CB1 deficiency into *Ptgs1^−/−^* mice to examine their parturition status. It was exciting to see that the loss of CB1 overrides COX-1 deficiency-induced delayed parturition (**Figure 5A**) and remarkably improves the survival rate of newborn pups (**Figure 5B**). Similar observations were also noted in pregnant *Ptgs1^−/−^* mice receiving SR1, but not SR2, on days 14–18 (**Figure 5C & D**). These results suggest that CB1 signaling has a unique role in regulating normal parturition that is independent of COX-1-derived prostaglandin F_2α_, but CB1 deficiency can correct the effects produced by COX-1 deficiency. Recent evidence suggests that cyclooxygenases participate in oxidative metabolism of endocannabinoids, owing to their structural similarity to polyunsaturated fatty acids. For example, both anandamide and 2-arachidonoylglycerol can serve as substrates for COX-2 [42]–[44], and COX-1 [45] in the context of cell types and conditions. Moreover, there is evidence that endocannabinoids via CB1 can upregulate COX-2 expression and thus prostaglandin E_2_ production in human gestational membranes during late pregnancy [46]. It remains to be determined whether COX-1 deficiency induced delayed parturition is associated with aberrant cannabinoid-CB1 signaling in mice.

### CB1 deficiency induces aberrant CRH-driven endocrine activities leading to preterm labor in mice

**Figure 6:**
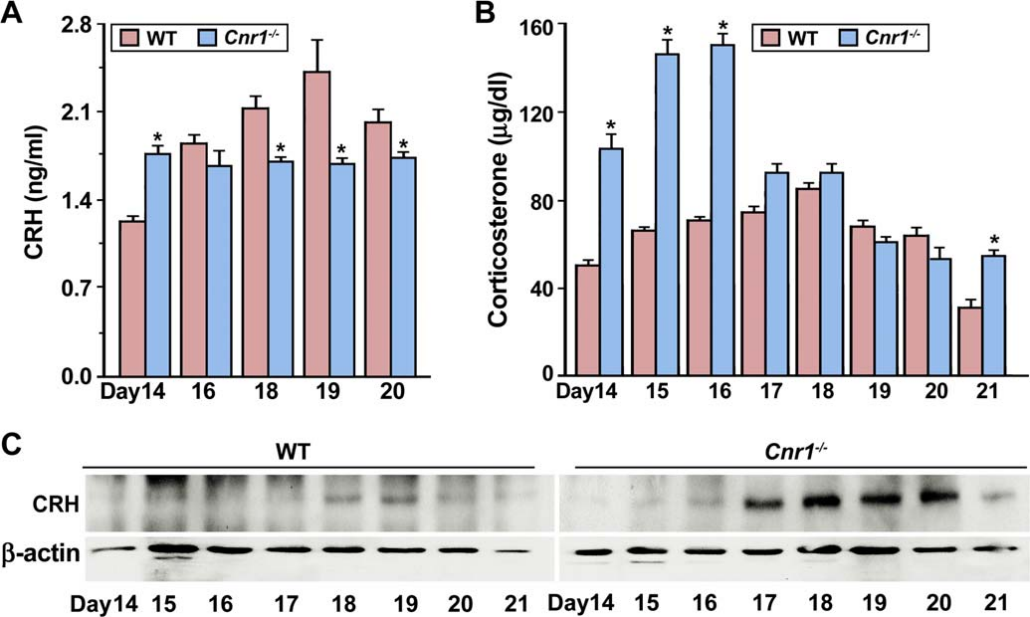
Aberrant levels of CRH and corticosterone from CB1 deficiency contribute to preterm birth in mice. (A & B) Circulating levels of CRH and corticosterone in pregnant WT and *Cnr1^−/−^* mice during late gestation (n = 4–5, *P<0.05). (C) Western blot analysis of CRH in WT and *Cnr1^−/−^* ovaries during late gestation, showing an early induction of ovarian CRH expression in mutant females.

Increasing evidence suggests that the endocannabinoid system influences the secretion of CRH [47], [48], which serves as a clock regulating the length of human pregnancy [25], [26]. To further reveal underlying causes of preterm birth in *Cnr1^−/−^* mice, we examined the status of CRH levels during late pregnancy. As illustrated in **Figure 6A**, while circulating CRH levels peaked on day 19 of pregnancy proceeding the day of labor onset in WT females, an aberrant CRH secretion pattern was noted in mutant females; the levels showed an early rise on day 14 and thereafter remained steady through day 20 in *Cnr1* ^−/−^ females. This early rise in CRH levels in peripheral circulation could be due to dysregulation of the hypothalamus-adrenal axis with an enhanced circadian drive on this stress-related axis in *Cnr1* ^−/−^ mice as previously demonstrated [49]. In fact, we observed significant increases in circulating corticosterone (CTS) levels on days 14–16 of pregnancy in mutant females compared with WT females (**Figure 6B**). These observations collectively point toward the concept that CB1 signaling is crucial for maintaining normal CRH-CTS activities prior to the onset of parturition in mice.

**Figure 7:**
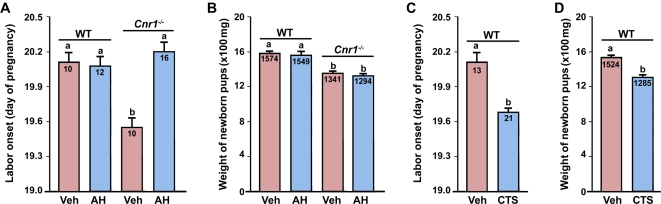
Pharmacological silencing of CRH activities by Antalarmin hydrochloride (AH) on days 15–17 restores normal labor in *Cnr1^−/−^* mice with little effects on fetal birth weights (A & B), while enhanced corticosterone (CTS) activity on days 14–18 induces preterm birth with impaired fetal growth in wild-type (WT) mice (C & D). Numbers within bars indicate the number of mice examined in panels A and C. The average fetal weights (mg) are shown in panels B and D. The bars with different letters are significantly different (P<0.01).

Moreover, as seen for CB1 (**Figure 2B**), immunostaining showed the presence of CRH and its type I receptor (CRH-RI) in WT day 18 corpora lutea (**Figure S3**), suggesting potential interactions of CB1 and CRH signaling within the ovary prior to parturition. In fact, we noted an early induction of ovarian CRH expression in *Cnr1^−/−^* mice with preterm labor (**Figure 6C**), further supporting that an altered CRH activity is one cause of preterm birth in mice missing CB1. Since there is evidence that CRH interferes with hypothalamic-pituitary-gonadal axis function by acting directly at the ovarian level [50], [51], we speculate that CB1 deficiency-induced aberrant CRH signaling is a potential cause of abnormal P_4_ and E_2_ secretion in *Cnr1* ^−/−^ ovary. However, it is to be noted that while the ovary as the primary site of P_4_ synthesis in pregnant mice contributes to CRH secretion, CRH like P_4_ is mainly produced by the placenta during late gestation in humans [47]. Regardless of the site of origin of CRH in various species, the physiological relevance of CRH-CTS axis in the onset of normal labor in mice is further supported by our observations that Antalarmin hydrochloride (AH), a CRH-RI selective antagonist, restored normal parturition in *Cnr1^−/−^* mice with little effects on fetal birth weights when females were treated on days 15–17 of pregnancy (**Figure 7A & B**). In contrast, elevated levels of CTS imposed by exogenous administration induced preterm birth with reduced fetal weights in WT females (**Figure 7C & D**), similar to labor defects in *Cnr1^−/−^* mice.

Collectively, we provide here multiple lines of evidence that signaling via CB1 is crucial for the normal onset of labor; its absence induces preterm birth via altering ovarian steroid synthesis and degradation, and CRH-CTS activities. These novel findings have high clinical relevance, since increases in circulating levels of anandamide are associated with labor onset and duration in women [23] and that CRH controls the length of human pregnancy [25], [26]. This is especially a concern in the light of little change in high preterm birth rates during the past 30 years [27], [28], and the use of a selective CB1 antagonist in Europe as an anti-obesity drug [52], [53]. There is evidence that polymorphism of the *Cnr1* gene occurs in humans [54] and is associated with variable drug dependency [55]. It remains to be seen whether preterm labor in women is associated with *Cnr1* gene polymorphism or mutation.

## Materials and Methods

### Animal models

All experiments were conducted in accordance with National Institutes of Health guidelines and were approved by the Intuitional Animal Care and Use Committee. *Cnr1*, *Cnr2* and *Ptgs1* mutant mice on C57BL/6J/129 mixed genetic background were generated as previously described [41], [56], [57]. *Cnr1^−/−^ and Ptgs1^−/−^* mice were cross-bred to generate *Cnr1^−/−^/Ptgs1^−/−^* double null mice. Females were mated with fertile males of the same strain to induce pregnancy. The presence of a vaginal plug was considered day 1 of pregnancy. Parturition events were monitored on days 19–21. The time of parturition was defined as complete delivery of pups.

### In vivo delivery of drugs

To explore the consequences of pharmacological silencing of CB1 receptors on the onset of parturition, pregnant females received daily subcutaneous administration of SR141716, a CB1 selective antagonist or SR144528, a CB2 selective antagonist at a dose of each 10 mg/Kg·BW, respectively on days 14–18 of pregnancy. To examine the effects of neutralizing CRH activities on the onset of labor, pregnant mice were treated daily with a CRH type I receptor selective antagonist Antalarmin hydrochloride at a dose of 25 mg/Kg·BW via subcutaneous injections on days 15–17. Pregnant wild-type mice treated with corticosterone at a dose of 40 mg/Kg·BW via subcutaneous injections on days 14–18 were examined to see the effects of enhanced corticosterone activity on parturition events. While SR141716 and SR144528 were obtained from National Institute on Drug Abuse, Antalarmin hydrochloride and corticosterone were obtained from Sigma. Drugs were dissolved in Triolein/ethanol (4∶1). Gestation length was measured by monitoring the parturition status on days 19–21.

### In situ hybridization

In situ hybridization of *Cnr1* in the brain was performed as previously described [58]. Briefly, frozen sections (10 µm) were mounted onto poly-L-lysine-coated slides and fixed in 4% paraformaldehyde solution in PBS at 4°C. After prehybridization, sections were hybridized at 45°C for 4 h in 50% formamide buffer containing ^35^S-labeled sense or antisense cRNA probes. After hybridization, sections were incubated with RNase A (20 µg/ml) at 37°C for 20 min, and RNase A-resistant hybrids were detected by autoradiography using Kodak NTB-2 liquid emulsion. Sections were poststained with hematoxylin and eosin. Sections hybridized with a sense probe did not show any positive signals and served as controls.

### Immunohistochemistry

Immunolocalization of CB1 in the pituitary, ovary and uterus were performed in formalin-fixed paraffin embedded sections using specific antibodies to CB1 receptor (Santa Cruz). A Histostain-Plus (DAB) kit (Invitrogen) was used to visualize the antigen.

### Assay P_4_, E_2_, corticosterone and CRH

Blood samples from WT and *Cnr1^−/−^*mice were collected on days 14–21 of pregnancy. Serum was separated by centrifugation (3,000 rpm for 15 min) and stored at −80°C until assayed. P_4_, E_2_, corticosterone levels were measured by radioimmunoassay by the UVA Center for Research in Reproduction Ligand Assay and Analysis Core. CRH was analyzed using an Enzyme Immuno Assay (EIA) kit purchased from Phoenix Pharmaceuticals INC following the manufacture's protocol.

### Statistical analyses

Data are expressed as means±s.e.m. Statistical comparisons between two experimental groups were determined by Student's t-test. A P value of less than 0.05 is considered significantly different.

## Supporting Information

Figure S1 Inter-crossing of Cnr1−/− females with wild-type (WT) males fails to correct preterm labor phenotype in Cnr1−/− females bearing heterozygous embryos (A), although fetal weights of heterozygous embryos were comparable to those of WT at birth (B). Data are means±SEM. Numbers within bars in panel (A) indicate the number of mice examined. The average fetal weights (mg) are shown in panel B. The bars with different letters are significantly different (Student t-test, P<0.01).
(1.35 MB TIF)

Figure S2 A single subcutaneous injection of progesterone (P4, 1 mg/mouse) on day 18 of pregnancy restores normal parturition (A) and fetal development at term (B) in Cnr1−/− mice. Mice receiving the same volume of sesame oil served as controls. Data are means±SEM. Numbers within bars in panel (A) indicate the number of mice examined. The average fetal weights (mg) are shown in panel B. The bars with different letters are significantly different (Student t-test, P<0.01).
(2.62 MB TIF)

Figure S3 Immunolocalization of CRH and CRH-RI in WT pregnant day 18 ovaries. Bar, 100 µm. CL, corpus luteum; f, follicle; Hip: hippocampus; o, oocyte.
(3.44 MB TIF)
