# Supplementary figures and images for: Loss of Cannabinoid Receptor CB1 Induces Preterm Birth
Source: PLoS One. 2008 Oct 3;3(10):e3320. doi: 10.1371/journal.pone.0003320 (PMC2553193; doi:10.1371/journal.pone.0003320)

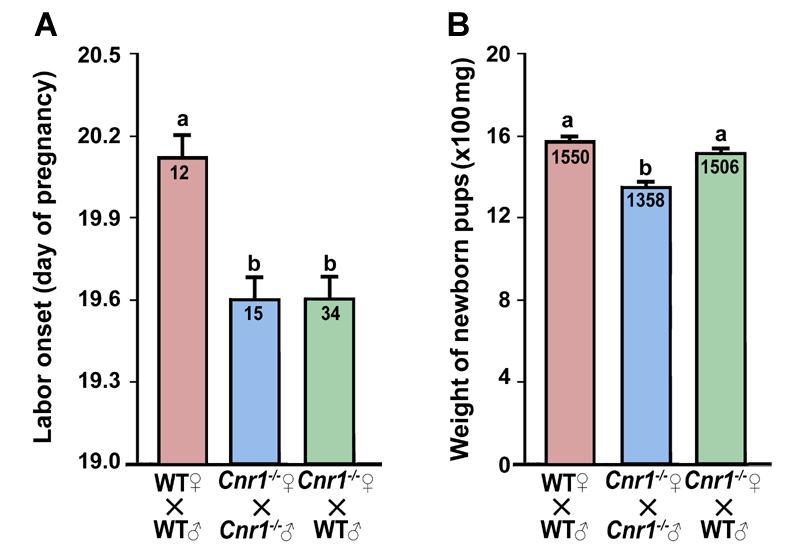

Supplement: Figure S1 — Inter-crossing of Cnr1−/− females with wild-type (WT) males fails to correct preterm labor phenotype in Cnr1−/− females bearing heterozygous embryos (A), although fetal weights of heterozygous embryos were comparable to those of WT at birth (B). Data are means±SEM. Numbers within bars in panel (A) indicate the number of mice examined. The average fetal weights (mg) are shown in panel B. The bars with different letters are significantly different (Student t-test, P<0.01). (1.35 MB TIF) [file pone.0003320.s001.tif]

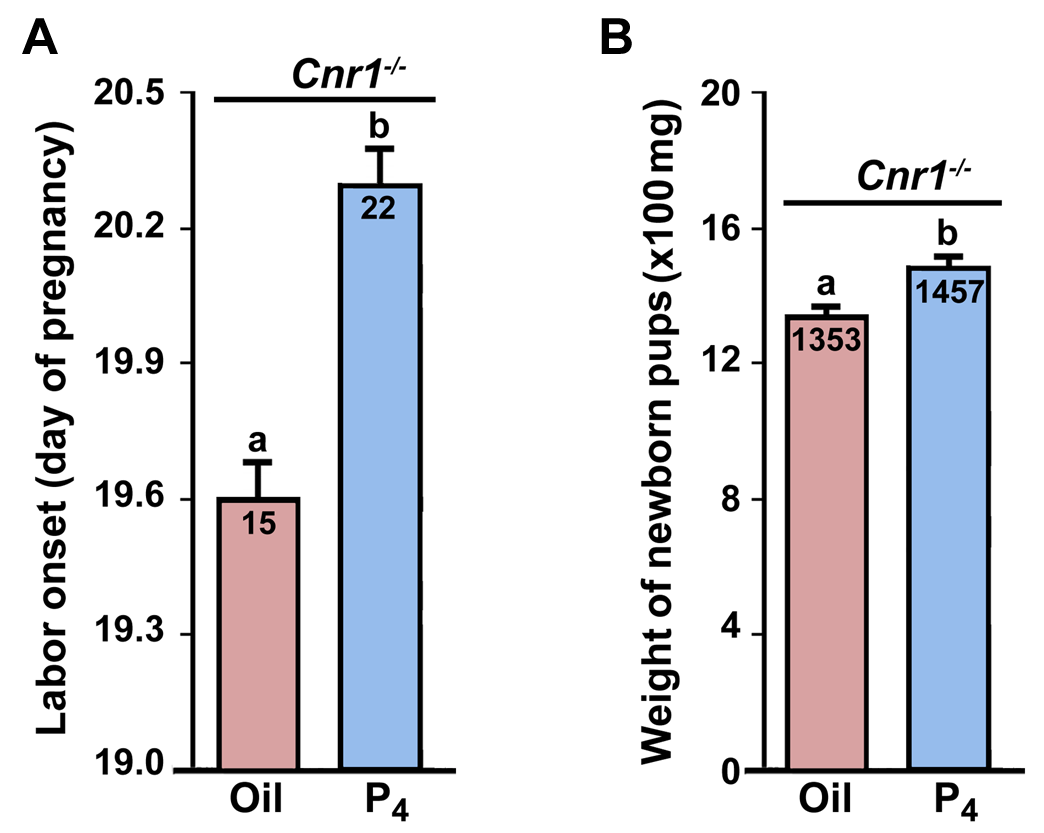

Supplement: Figure S2 — A single subcutaneous injection of progesterone (P4, 1 mg/mouse) on day 18 of pregnancy restores normal parturition (A) and fetal development at term (B) in Cnr1−/− mice. Mice receiving the same volume of sesame oil served as controls. Data are means±SEM. Numbers within bars in panel (A) indicate the number of mice examined. The average fetal weights (mg) are shown in panel B. The bars with different letters are significantly different (Student t-test, P<0.01). (2.62 MB TIF) [file pone.0003320.s002.tif]

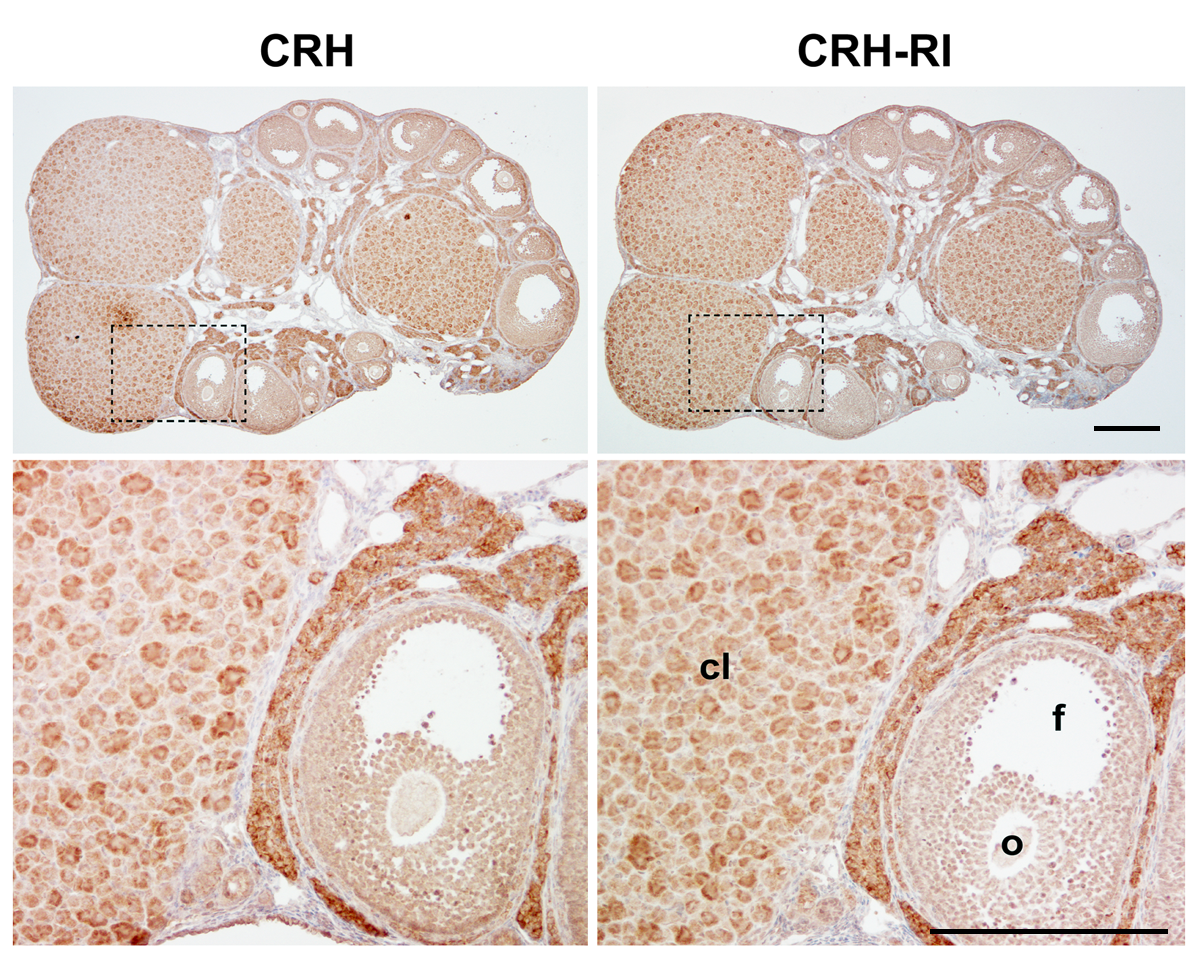

Supplement: Figure S3 — Immunolocalization of CRH and CRH-RI in WT pregnant day 18 ovaries. Bar, 100 µm. CL, corpus luteum; f, follicle; Hip: hippocampus; o, oocyte. (3.44 MB TIF) [file pone.0003320.s003.tif]
